# Exams at classroom have bidirectional effects on the long-term memory of an unrelated graphical task

**DOI:** 10.1038/s41539-018-0036-7

**Published:** 2018-11-06

**Authors:** P. Lopes da Cunha, D. Ramirez Butavand, L. B. Chisari, F. Ballarini, H. Viola

**Affiliations:** 1 Facultad de Medicina, Universidad de Buenos Aires, Buenos Aires, Argentina; 2 Laboratorio de Memoria, Instituto de Biología Celular y Neurociencias “Dr Eduardo De Robertis” (IBCN), CONICET- Universidad de Buenos Aires, Buenos Aires, Argentina; 3 Laboratorio de Neurociencia Traslacional, Instituto de Biología Celular y Neurociencias “Dr Eduardo De Robertis” (IBCN), CONICET- Universidad de Buenos Aires, Buenos Aires, Argentina; 4 Universidad de Buenos Aires, Facultad de Ciencias Exactas y Naturales. Departamento de Fisiología, Biología Molecular y Celular “Dr. Héctor Maldonado” (FBMC), Buenos Aires, Argentina; 5 Present Address: Instituto de Neurociencia Cognitiva y Traslacional (INCyT), Universidad de Favaloro, Buenos Aires, Argentina

## Abstract

The influence of a given event on long-term memory formation of another one has been a relevant topic of study in the neuroscience field in recent years. Students at school learn contents which are usually tested in exam format. However, exam elevates the arousal state of the students acting as a mild stressor that could influence another memory formation ongoing process. Thus, in this study we examine in high school students the effect of exams on long-term retention of unrelated information, learned at different times before or after the exams. Our results show that exams are not innocuous and that they could improve or reduce the retention of temporarily associated content. These effects did not show gender differences. Our findings should alert teachers about the side effects of exams on the learning of other content within the same school day.

## Introduction

Learning and memory are cognitive brain functions of fundamental importance in our educational context. The formation of a stable memory trace goes through a gradual process susceptible to factors that could improve or impair it.^1^ Some novel, arousing or stressing situations could impact on memory formation and their effects depend on their duration, intensity and moment of application.^2^ In that sense, exams constitute mild stressors used as routine tools for assessment means, and their potential effects on temporally close learning has not been evaluated so far. In the present work, we studied the effect of an exam on long-term retention of an unrelated graphical memory task acquired at the classroom.

## Results

Students were allowed to copy a figure at the training day (TR) and 24 h later they were asked to draw it again. A memory index was calculated. The first analysis was to assess the children performance on TR day. We noticed that around 85% of the participants copied the figure completely, whereas 12% missed copying 1 element and 3% missed copying 2 elements. However, when we compared the memory index between participants who copied the figure completely, those who missed 1 element and those who missed 2, the one-way ANOVA did not reveal significant differences between these three groups, neither for weak controls (*F*_(2,53)_ = 1.24, *p* = 0.30) nor for strong controls (*F*_(2,55)_ = 0.04, *p* = 0.96).

**Fig. 1:**
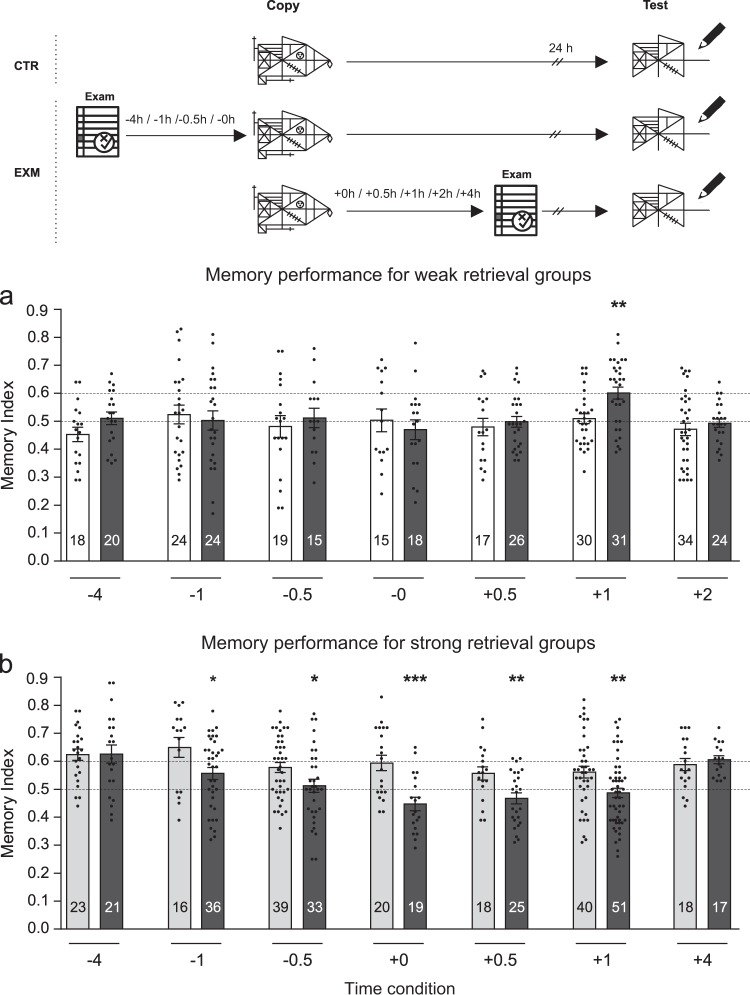
Exam effects on unrelated graphical long-term memory could be beneficial or deleterious. A schematic representation of the experimental protocol is presented on the top of the figure: students were asked to copy Rey Osterrieth´s figure and they had or not (CTR) an exam before or after it. The figure copy is time zero and the time condition described for the exams are relative to it and expressed in hours. LTM of this figure was tested 24 h later. **a** Memory Index is shown as mean ± SEM for weak retrieval CTR groups (CTRw, white bars) and for that groups which had one exam at different times around copying of the figure (EXM groups, black bars). **b** Memory Index is shown as mean ± SEM for strong retrieval CTR groups (CTRs, light gray bars) and for that groups which had one exam at different times around copying of the figure (EXM groups, black bars). In all cases the number of participants is written in each bar. Student’s *t*-test, CTR vs. corresponding EXM group, *p < 0.05, **p < 0.01, ***p < 0.001

Figure 1a shows the performance in the graphical LTM test for students from CTRw, which obtained a Memory Index of about 0.5 and significantly less than 0.6 (One-sample *t*-test against 0.5 value, *p* > 0.05; against 0.6 value *p* < 0.05; for all CTRw groups). We observed an improvement of the Memory Index for students who experienced the exam 1 h after the copying of the figure in the TR day (Student’s *t*-test; EXM Condition +1 h vs CTRw*, t* = 3.30, *p* < 0.01). The other EXM groups, whose students had the exam previous to the copying of the figure (−4, −1, −0.5, or −0 h) or at other times after it (+0.5 h or +2 h), showed similar Memory Indices in comparison to their respective control groups (EXM vs. respective CTRw, *p* > 0.05). Figure 1b shows the performance in the graphical LTM test from CTRs, which obtained a Memory Index of about 0.6 and significantly greater than 0.5 (One-sample *t*-test: against 0.6 value *p* > 0.05; against 0.5 value *p* < 0.05; for all CTRs groups). In contrast to the improvement in memory retrieval observed in Fig. 1a, the presence of an exam either before (−1 or −0.5 h) or after (+0, +0.5, or +1 h) the copying of the figure, significantly decreased the memory index of the EXM groups with respect to their corresponding CTRs groups (Student’s *t*-test; EXM vs respective CTRs, at condition −1 h: *t* = 2.33, *p* < 0.05; −0.5 h: *t* = 2.24, *p* < 0.05; +0 h: *t* = 3.98, *p* < 0.001; +0.5 h: *t* = 2.92, *p* < 0.01; +1 h: *t* = 2.78, *p* < 0.01). However, when the exam was taken 4 h previous to the figure copy or 4 h after it, the impairment of LTM was not observed (EXM vs respective CTRs, *p* > 0.05).

**Fig. 2:**
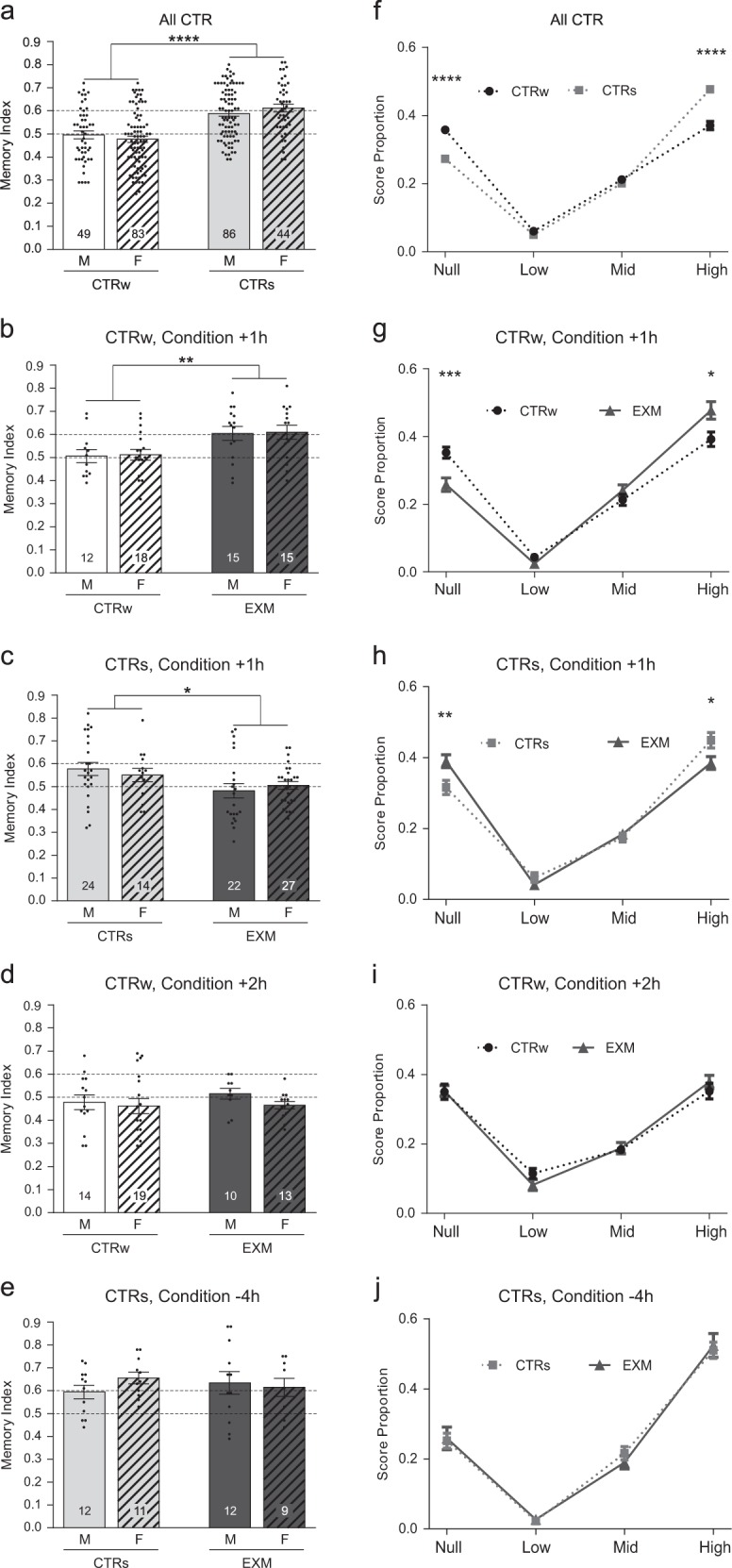
Sex differences and proportion of item scores for graphical memory. **a**–**e** Memory index is shown as mean ± SEM for different groups in which masculine (M, flat bars) and feminine (F, hatched bars) students were analyzed separately. We show data for all CTRw (white bars) and CTRs (light gray bars) **a**, EXM groups (black bars) that had an exam 1 h after the copying of the figure (EXM, Condition +1 h) in both weak **b** and strong **c** retrieval students groups and also for those groups that had an exam 2 h after the copying of the figure (EXM, Condition +2 h) or 4 h before it (EXM, Condition -4h) in weak **d** and strong **e** retrieval students groups, respectively. Two-way ANOVA, *p < 0.05, **p < 0.01, ****p < 0.0001. **f**–**j** Score proportion is shown as mean ± SEM for different groups in which item scores (null, low, mid, and high) was analyzed. We compare the profile of CTRw (black dotted line with circles) and CTRs (gray dotted line with squares) **f**, EXM in Condition +1 h (black line with triangles) with its respective CTRw **g** or CTRs **h**, EXM in Condition +2 h group with its respective CTRw **i** and EXM in Condition -4h with its respective CTRs **j**. Student’s *t*-test. *p < 0.05, **p < 0.01, ***p < 0.001, ****p < 0.0001

Then, we analyzed possible sex differences in memory index using two-way analysis of variance (ANOVA). In Fig. 2a we assessed differences applying control group (CTRw/CTRs) x gender (M/F) ANOVA. We found a significant main effect of control type, *F*_(1,258)_ = 57.13, *p* < 0.0001, but no main effect of gender, *F*_(1, 258)_ = 0.02, *p* = 0.88, neither significant interaction, *F*_(1, 258)_ = 1.97, *p* = 0.16. These results showed that beyond the differences between CTRw and CTRs no significant differences were observed between masculine (M) and feminine (F) students in each one. The following two-way ANOVA analysis was made considering group condition (EXM/CTRw or EXM/CTRs) x gender (M/F) for different time point evaluated. The increment in the Memory Index induced by the exam 1 h after the copying of figure observed in weak retrieval students, was similar for M and F, since the analysis only revealed a main effect of exam, *F*_(1, 56)_ = 11.89, *p* < 0.01 (gender: *F*_(1, 56)_ = 0.04, *p* = 0.84; interaction: *F*_(1, 56)_ = 7.75 e−5, *p* = 0.99; Fig. 2b). Moreover, the decrement induced by the exam 1 h after the copying of figure in strong retrieval students was similar for M and F too (exam: *F*_(1, 83)_ = 6.61, *p* < 0.05, gender: *F*_(1, 83)_ = 3.00 e−3, *p* = 0.96, interaction: *F*_(1, 83)_ = 0.83, *p* = 0.37; Fig. 2c). Also, at those times in which the exams had no effect, for example exam 2 h after copying of the figure in weak retrieval students (Fig. 2d) or exam 4 h before it in strong retrieval students (Fig. 2e), the Memory Index did not show differences by gender. The analysis did not reveal any significant effect (at Condition +2 h in weak retrieval groups, exam: *F*_(1, 52)_ = 0.43, *p* = 0.52, gender: *F*_(1, 52)_ = 1.19, *p* = 0.28, interaction: *F*_(1, 52)_ = 0.30, *p* = 0.59; at Condition −4 h in strong retrieval groups, exam: *F*_(1, 40)_ = 1.78 e−4, *p* = 0.99, gender: *F*_(1, 40)_ = 0.30, *p* = 0.59, interaction: *F*_(1, 40)_ = 1.14, *p* = 0.29).

Finally, we analyzed the score proportion obtained in figure's test using Student's *t*-test. Figure 2f shows a decreased proportion of null score (*t* = 5.58, *p* < 0.0001) and an increment in items perfectly drawn (high score*; t* = 6.33, *p* < 0.0001) in CTRs compared to CTRw. No differences were observed in the low and mid scores (*p* > 0.05). This same profile was observed in weak retrieval students, when EXM Condition +1 h group was compared with their respective CTRw (null score*, t* = 3.62, *p* < 0.001; high score*, t* = 2.54, *p* < 0.05; low and mid scores *p* > 0.05; Fig. 2g). On the other hand, the decrement of memory index observed in strong retrieval students at EXM Condition +1 h compared with their controls, relayed on the increment of null items drawn in the test and in a decrement of items perfectly drawn (null score*, t* = 2.76, *p* < 0.01; high score*, t* = 2.28, *p* < 0.05; Fig. 2h). No differences were observed in the low and in mid scores (*p* > 0.05). These results suggest that the amount of items drawn and the number of items faithful drawing (both draw and place were correct) are parameters sensible to exams effects. Moreover, the analysis of score proportion in those cases in which memory index was not modified, did not show differences between groups (EXM Condition +2 h vs. CTRw: all scores *p* > 0.05, Fig. 2i; EXM Condition -4h vs CTRs**:** all scores *p* > 0.05, Fig. 2j).

## Discussion

Empirical evidence showing that retrieval practice enhances learning supports the use of exams in schools.^3^ However, the role of exams on surrounding unrelated learnings has not been studied. Our data show that the effect of an exam on an unrelated graphical LTM could be beneficial or deleterious. In the group of students expressing a weak graphical LTM, the exam improved it only if it was taken 1 h after the copying of Rey’s figure. This positive effect was similar (20–25% of the total control retention score) to the action of novelty previously reported by us in elementary school children.^4^ In both works figure activities were applied by researchers, which constituted a novel situation for all students, but we expect the effect of an outsider’s intrusion into the classroom to not be different for the students in control or exam groups. However, in the present work the arousal is caused by programmed exams, while in the Ballarini et al work, it was caused by a novel science lesson applied by a novel teacher. On the other hand, university students subjected to an emotionally arousing video after a lecture in psychology showed an enhancement in lecture LTM.^5^ In line with the present results, it was shown that strong experiences associated with a weak learning improved its LTM expression.^6^ In order to explore the effects of stress on the phases of episodic memory, a meta-analysis was recently performed with thousands of participants. It concluded that post-encoding stress improved memory unless the stressor occurred in a different physical context than the study materials.^7,8^

In general, post-learning stress in human beings enhances LTM, but when it take place before the learning, it shows inconsistent findings.^9^ Furthermore, the effect of pre-learning stress is often stronger for emotional compared with neutral learning material.^10^ When stress occurred prior to or during encoding it impaired memory, unless the study materials were directly related to the stressor and the delay between them was very short. In such cases, stress improved encoding.^7,11,12^

For strong retrieval populations of students, our study shows a negative exam effect on LTM graphical retention that only occurs when the interval between the tasks in the training day was up to one hour. A possible explanation for this fact is that the molecular processing triggered for both tasks could interact within a temporal time-window, while broader intervals than this could avoid this interaction. One cellular mechanism proposed to explain this late associative effect among stimuli or events is provided by the hypothesis of synaptic tagging and capture^13^ and its behavioral counterpart, the behavioral tagging.^14^

In a wider scenario, it is important to highlight that stress has a critical impact on the formation, retrieval and reactivation of memory, which is at the heart of our educational system. An integrative view on the effects of stress at classroom, both on students and teachers, was made by Vogel and Schwabe.^15^ With the aim of contributing to this field, our findings alert about exams’ influence on extrinsic memory processes taking place up to one hour before or after them. In conclusion, our data suggests educators should be attentive to their student's exams’ schedule, as this will modulate the retention of the contents of previous or later teaching.

## Methods

Six hundred and thirty one participants (ages 12–17 years-old) from six different schools in Buenos Aires, Argentina were tested. All students were naive to the procedure. This study was performed under the approval of the Ethical Committee of the Faculty of Medicine of the University of Buenos Aires. Procedures were reviewed and approved by the Head of each participating educational institution and a consent was signed by students’ parents. Students were allowed to withdraw from the study at any time without consequences.

We designed a task to test graphic memory based on Rey Osterrieth’s figure test.^4,16^ On TR day, this figure was shown to the whole course of students and each student was allowed to copy it on a blank paper for a 5 min period. This amount of time was enough to make the copy in its entirety. Once finished, drawings were handed in to the researchers. Long-term memory (LTM) of this figure was tested 24 h later by asking the students to draw it again on a blank paper during a 5 min interval. Rey Osterrieth’s figure copy and test activities were applied by the researchers. An individual retention score was calculated taking into account each of the 18 items of the figure, analyzing item location, accuracy, and organization according to Rey-Osterrieth’s scoring scheme.^17^ Briefly, when an item is not drawn, it has a zero score (null); if it is drawn in a wrong location and with mistakes it has a 0.5 score (low); if an item is in the right location but partially correct as well as in the wrong location but drawn without mistakes it has a score of 1 (mid). Only when an item is drawn correctly and placed properly, the score is 2 (high). So, for each student we calculated a memory index, which is the ratio between performance in drawing the figure on the test day and performance in drawing the figure on the learning day. Also, we quantified the amount of null, low, mid and high scores and we related them to the total amount of items to calculate the score proportion obtained for students in the figure test.

Students assigned to the exam group (EXM) had a single curricular written exam at classroom on TR day, previously established and occurring at some time before (between −4 and −0 h) or after (between +0 h and +4 h) the copying of the figure. The exams were applied by the corresponding teachers. Students of another course without exams at the copying’s day were assigned to the control groups (CTR). On the day of the test, none of the groups had exams.

We always assigned a priori a CTR group of students with its respective EXM condition group, being this pair of groups obtained at the same institution and containing students in comparable conditions (same age, with the copying occurring at the same day and time of the day). In some cases, in which schools had three class sections, one of these was assigned to CTR and the other two were assigned to different EXM condition groups. So, a given CTR course was always paired and compared with a specific EXM course.

Afterwards, we analyzed our data and found differences in the Memory Index (LTM) obtained from CTR groups. Some of them had high memory indices (“strong retrieval”, CTRs) while others CTR groups had low one (“weak retrieval”, CTRw). Within the same school, CTR groups were always belonged to the same control type. The CTRw groups were compared through one-way ANOVA and we did not find significant differences between them (*F*_(6, 150)_ = 0.74, *p* = 0.62). The same happened with the CTRs groups (*F*_(6, 167)_ = 1.72, *p* = 0.12). When we compared CTRw vs CTRs means, we found a significant difference (*p* < 0.001, *t* = 6.65), reason why these control groups were analyzed separately and compared with their corresponding EXM groups.

All information about schools, age range or gender of students, for all time conditions is shown in Supplementary table S1.

Student’s *t*-test or two-way analysis of variance (ANOVA) was conducted for analyze the data collected and size effect was calculated (see Supplementary table S2).

## Electronic supplementary material

Supplementary information

## Data Availability

The data that support the findings of this study are available from the corresponding author upon reasonable request.

## Electronic supplementary material

**Supplementary information** accompanies the paper on the *npj Science of Learning* website (10.1038/s41539-018-0036-7).
