# Supplementary material for: Exams at classroom have bidirectional effects on the long-term memory of an unrelated graphical task
Source: NPJ Sci Learn. 2018 Nov 6;3:19. doi: 10.1038/s41539-018-0036-7 (PMC6220208; doi:10.1038/s41539-018-0036-7)
Supplement: Supplementary file 1 — Supplementary information [file 41539_2018_36_MOESM1_ESM.pdf]

## Supplementary information

TABLE S1. Details of school, age range and gender for all time conditions.

| School | Private/Public | Courses | Mean Age | Female | Male | CTR/EXM | Condition (h) | Weak (W)/ Strong (S) |
|---|---|---|---|---|---|---|---|---|
| #1 | Private | A | 13 | 11 | 12 | CTR | -4 | S |
| | | B | 13 | 9 | 12 | EXM | -4 | S |
| | | C | 13 | 10 | 6 | CTR | -1 | S |
| | | D | 13 | 12 | 5 | EXM | -1 | S |
| | | E | 13 | 12 | 7 | EXM | -1 | S |
| | | F | 13 | 6 | 15 | CTR | 1 | S |
| | | G | 14 | 11 | 8 | EXM | 1 | S |
| #2 | Private | A | 14 | 0 | 19 | CTR | -0.5 | S |
| | | B | 14 | 0 | 17 | EXM | -0.5 | S |
| | | A | 14 | 0 | 20 | CTR | 0 | S |
| | | C | 14 | 0 | 26 | EXM | 0 | S |
| | | D | 15 | 0 | 18 | CTR | 4 | S |
| | | E | 16 | 0 | 11 | EXM | 4 | S |
| | | F | 16 | 0 | 6 | EXM | 4 | S |
| #3 | Public | A | 16 | 10 | 10 | CTR | -0.5 | S |
| | | B | 16 | 10 | 6 | EXM | -0.5 | S |
| | | C | 13 | 8 | 9 | CTR | 0.5 | S |
| | | D | 13 | 18 | 7 | EXM | 0.5 | S |
| | | C | 13 | 9 | 9 | CTR | 1 | S |
| | | E | 13 | 18 | 14 | EXM | 1 | S |
| #4 | Private | A | 13 | 17 | 1 | CTR | -4 | W |
| | | B | 13 | 7 | 13 | EXM | -4 | W |
| | | C | 16 | 14 | 5 | CTR | -0.5 | W |
| | | D | 16 | 8 | 6 | EXM | -0.5 | W |
| | | E | 15 | 10 | 5 | CTR | 0 | W |
| | | F | 14 | 11 | 7 | EXM | 0 | W |
| | | G | 17 | 13 | 4 | CTR | 1 | W |
| | | H | 17 | 10 | 10 | EXM | 1 | W |

| | | | | | | | | |
|---|---|---|---|---|---|---|---|---|
| #5 | Private | A | 14 | 9 | 15 | CTR | -1 | W |
| | | B | 14 | 16 | 8 | EXM | -1 | W |
| | | C | 13 | 13 | 4 | CTR | 0.5 | W |
| | | D | 13 | 13 | 13 | EXM | 0.5 | W |
| | | C | 13 | 13 | 4 | CTR | 2 | W |
| | | E | 13 | 10 | 4 | EXM | 2 | W |
| | | F | 17 | 6 | 10 | CTR | 2 | W |
| | | G | 17 | 4 | 6 | EXM | 2 | W |
| #6 | Private | A | 17 | 5 | 8 | CTR | 1 | W |
| | | B | 17 | 5 | 5 | EXM | 1 | W |

TABLE S2. Relevant statistical information including size effect.

| Groups | Figure | Statistic | p | Cohen's d |
|---|---|---|---|---|
| CTRw vs EXM+1 | 1a | t=3.30 | 0.002 | 0.845 |
| CTRs vs EXM-1 | 1b | t=2.33 | 0.024 | 0.701 |
| CTRs vs EXM-0.5 | 1b | t=2.24 | 0.028 | 0.531 |
| CTRs vs EXM+0 | 1b | t=3.98 | <0.001 | 1.274 |
| CTRs vs EXM+0.5 | 1b | t=2.92 | 0.006 | 0.904 |
| CTRs vs EXM+1 | 1b | t=2.78 | 0.007 | 0.587 |
| CTRw vs CTRs | 2a | F of Control Factor: 57.13 | <0.0001 | 0.938 |
| | | F of Gender Factor:0.02 | 0.8827 | |
| | | F of Interaction: 1.97 | 0.1622 | |
| CTRw vs EXM+1 | 2b | F of Condition Factor: 11.89 | 0.0011 | 0.906 |
| | | F of Gender Factor:0.04 | 0.8448 | |
| | | F of Interaction: 7.75e-5 | 0.9930 | |
| CTRs vs EXM+1 | 2c | F of Condition Factor: 6.611 | 0.0119 | 0.562 |
| | | F of Gender Factor:0.003 | 0.9564 | |
| | | F of Interaction: 0.825 | 0.3663 | |
| Null: CTRw vs. CTRs | 2f | t=5.58 | <0.0001 | 0.673 |
| High: CTRw vs. CTRs | 2f | t=6.33 | <0.0001 | 0.764 |
| Null: CTRw vs. EXM | 2g | t=3.62 | 0.0006 | 0.927 |
| High: CTRw vs. EXM | 2g | t=2.54 | 0.0138 | 0.651 |
| Null: CTRs vs. EXM | 2h | t=2.76 | 0.007 | 0.583 |
| High: CTRs vs. EXM | 2h | t=2.28 | 0.0248 | 0.482 |
